# Design of highly stabilized nanocomposite inks based on biodegradable polymer-matrix and gold nanoparticles for Inkjet Printing

**DOI:** 10.1038/s41598-019-52314-2

**Published:** 2019-11-06

**Authors:** Belen Begines, Ana Alcudia, Raul Aguilera-Velazquez, Guillermo Martinez, Yinfeng He, Ricky Wildman, Maria-Jesus Sayagues, Aila Jimenez-Ruiz, Rafael Prado-Gotor

**Affiliations:** 1 Department of Organic and Medicinal Chemistry, School of Pharmacy, University of Seville, Seville, 41012 Spain; 2 Centre for Additive Manufacturing, Faculty of Engineering, University of Nottingham, Nottingham, NG7 2RD United Kingdom; 3 Material Science Institute of Seville, CSIC/US, Seville, 41092 Spain; 4 Department of Physical Chemistry, School of Pharmacy, University of Seville, Seville, 41012 Spain

**Keywords:** Chemistry, Biomedical engineering, Sensors and biosensors

## Abstract

Nowadays there is a worldwide growing interest in the Inkjet Printing technology owing to its potentially high levels of geometrical complexity, personalization and resolution. There is also social concern about usage, disposal and accumulation of plastic materials. In this work, it is shown that sugar-based biodegradable polyurethane polymers exhibit outstanding properties as polymer-matrix for gold nanoparticles composites. These materials could reach exceptional stabilization levels, and demonstrated potential as novel robust inks for Inkjet based Printing. Furthermore, a physical comparison among different polymers is discussed based on stability and printability experiments to search for the best ink candidate. The University of Seville logo was printed by employing those inks, and the presence of gold was confirmed by ToF-SIMS. This approach has the potential to open new routes and applications for fabrication of enhanced biomedical nanometallic-sensors using stabilized AuNP.

## Introduction

In recent years, Additive Manufacturing (AM), also commonly known as 3D Printing, has been recognized as an enabling technology for applications that require intricate geometries or personalization^1,2^. Its layer by layer based fabrication allows direct production from a CAD design, reducing overhead costs for small batch bespoke production when compared with traditional manufacturing methods. Among the seven categories of 3D printing techniques proposed by the American Society for Testing and Materials (ASTM), Material jetting (MJ)^3^ —also known as Inkjet Printing (IJP) and defined as *an additive manufacturing process in which droplets of build materials are selectively deposited*— is gaining high attention for its unique combination of advantages including its capabilities for multi-materials, production resolution and scalability. Among these, multi-materials printing opens the door to the direct fabrication of multi-functional components in a single step, revolutionizing industries^4–6^ in the fields, for example, of Electronics or Biomedicine. Although IJP as a 3D Printing technique has been known since the 90 s, limited materials availability has restricted its use until the last decade. However, the development of new materials/applications for this technique has recently become a priority for many researchers^7–13^. In this sense, many interdisciplinary studies towards developing efficient engineered entities such as nanomaterials have been conducted. Nano-particulate materials, and gold nanoparticles (AuNP) specifically^14^, are highly promising owing to their biocompatibility. They have been employed in fields such as diagnostics^15,16^, as revealed by flow immunoassays used with biacore focused on measuring biomolecular interactions, including protein-protein interactions or small molecule/fragment-protein interactions; catalysis^17^, such as the aerobic oxidative processes; or therapeutic agent delivery^18,19^ in the case of an appropriated coating of gold nano-structures bearing anti-fouling polymers or targeting agents to enhanced drug delivery, taking advantage of the enhanced permeability and retention (EPR) and the improvement of the PEGylation process. However, one of the most explored applications of AuNP systems is their use in nanometallic sensors^20–23^, for instance, the colorimetric sensors to analyze food in bromatology. They also show great potential in areas such as electronics or nanobioelectronics^24,25^, owing to their potential to form low resistanse conductor patterns. Taking advantage of this property, AuNP systems have been applied for electrical and electrochemical sensing^22^. Thus, Wohltjen and Snow^26^ used the variation in the conductance of a AuNP based chemoresistor to determine the presence of toluene and tetrachloroethylene. By modifying the ligands, *Kim et al*.^27^ proved the response towards 1-propanol, acetone and cyclohexane. Likewise, electrochemical detection of numerous small biomolecules, toxic chemicals and drugs has been explored^18^. For example, Mattiasson *et al*. developed a glucose biosensor based on a concanavalin-AuNP system and a polytyramine-modified gold electrode^28^. *Singh et al*. used a fullerene-C(60)-modified gold electrode to determine levels of prednisolone down to 26 nM^29^. Furthermore, electrochemical AuNP systems have been widely employed for the recognition of tumor cells^30,31^ and cancer biomarkers, such as α-fetoprotein^32–34^, carcinoma antigen 125^35^, carbohydrate antigen 19−9^36^, carbohydrate antigen 125^37^, prostate-specific antigen^38^, or mammary cancer 15−3 antigen^39^. Likewise, different types of biomarkers have been determined with the use of AuNP systems, including cholera toxin^40^, vascular endothelial growth factors^41^, *Salmonella typhi* antigen^42^, foodborne pathogen *Escherichia coli* O157:H7^43^ or markers for acute myocardial infarction^44,45^, among others. Most of these electrical or electrochemical sensing systems are based not only in the use of AuNP-based systems but also in the utilisation of Au electrods. Therefore, the advance in the personalisation and multi-material direct fabrication of sensors requires the optimisation of gold containing inks. Currently, the best approach to inkjet print metals is its application as nanoparticles. However, the use of inks for inkjet printing based exclusively on metallic nanoparticles has shown poor commercial results due to the low stability of the ink. At this point, new approaches to ink fabrication geared towards those applications need to be explored. One of such venues can be the combination of gold nanoparticles and suitable stabilizing agents, such as natural or synthesized polymers, that are able to effectively encase gold nanoparticles while retaining their own ink-suitable plastic properties.

Significantly, in the last several decades the constant accumulation of polymer plastic materials obtained from fossil oils and the contamination generated by its industry is causing a world-wide concern about environmental damage and its future implications^46–49^. Among other potential solutions for reduction of long-lasting residues, the replacement of petroleum-derived polymers by new polymeric materials based on renewable resources is been widely investigated^50,51^. For this aim, the use of carbohydrates as raw materials for the synthesis of *comb*-like polymers is highly recommended due to their low cost and widespread availability, since they are present in all living organisms or their products. Likewise, another important characteristic inherent to carbohydrates nature is their potential for derivatization, leading to a large range of structures and thus applications^52,53^. In this sense, *comb*-like polymers are a special type of graft-polymers with half-way characteristics between linear and branched polymers^54,55^. This type of polymeric materials has been already investigated for the synthesis of AuNP^56,57^. In particular, according to Deng *et al*., the use of *comb*-like polymers in combination with metal nano-structures leads to an unique side-chain crystallization phenomenon that allows a reduction of the distance between adjacent nanoparticles. They also were found to prevent AuNP aggregation and enhance stability. Thus, the multifunctional-terminated group could bond more than one AuNP on a single polymeric unit forming AuNP/polymer macrostructures. Furthermore, this AuNP/polymer nanocomposite could further self-assemble by hydrophilic/hydrophobic interactions. Hence, these characteristics allowed the formation of AuNP with a high level of packing observed in TEM micrography. Considering the excellent biocompability of gold nanoparticles with different kinds of biopolymers, they provide an endless source of possibilities and applications in different fields of science^13^. As an example, described in our lab, it suffices to quote the use of gold nano-structures as crosslinkers to achieve chitosan nanocapsule networks^58^, as colorimetric sensors^59^ and for DNA delivery^60^.

In our search for new gold containing inks, we took advantage of the great potential of *comb*-like polymers to stabilize AuNP and the biocompatibility and low contamination levels offered by carbohydrates based polyurethanes. In this sense, this work aimed at confirming that sugar-based *comb*-like polyurethanes (PU) derived from sustainable precursors, recently reported by our synthesis laboratory^52^, could not only be suitable for the preparation of AuNP embedded in these materials, but strong candidates for testing as novel metallic materials for ink jet printing in the 3D-printing process. In this work, different structures of *comb*-like PU are explored to generate three nanocomposites, with the aim of developing gold-based inks that can be employed in place of the unprotected nanoparticles that, as mentioned before, are not suitable for an IJP process. The printing stability was investigated using inkjet printing and showed stable processability. Finally, the success in the usage of biodegradable *comb*-like copolyurethanes as AuNP stabilizing matrices was demonstrated by the IJP of the University of Seville logo.

## Methods

### Synthesis of polymer-encased AuNPs

**Figure 1:**
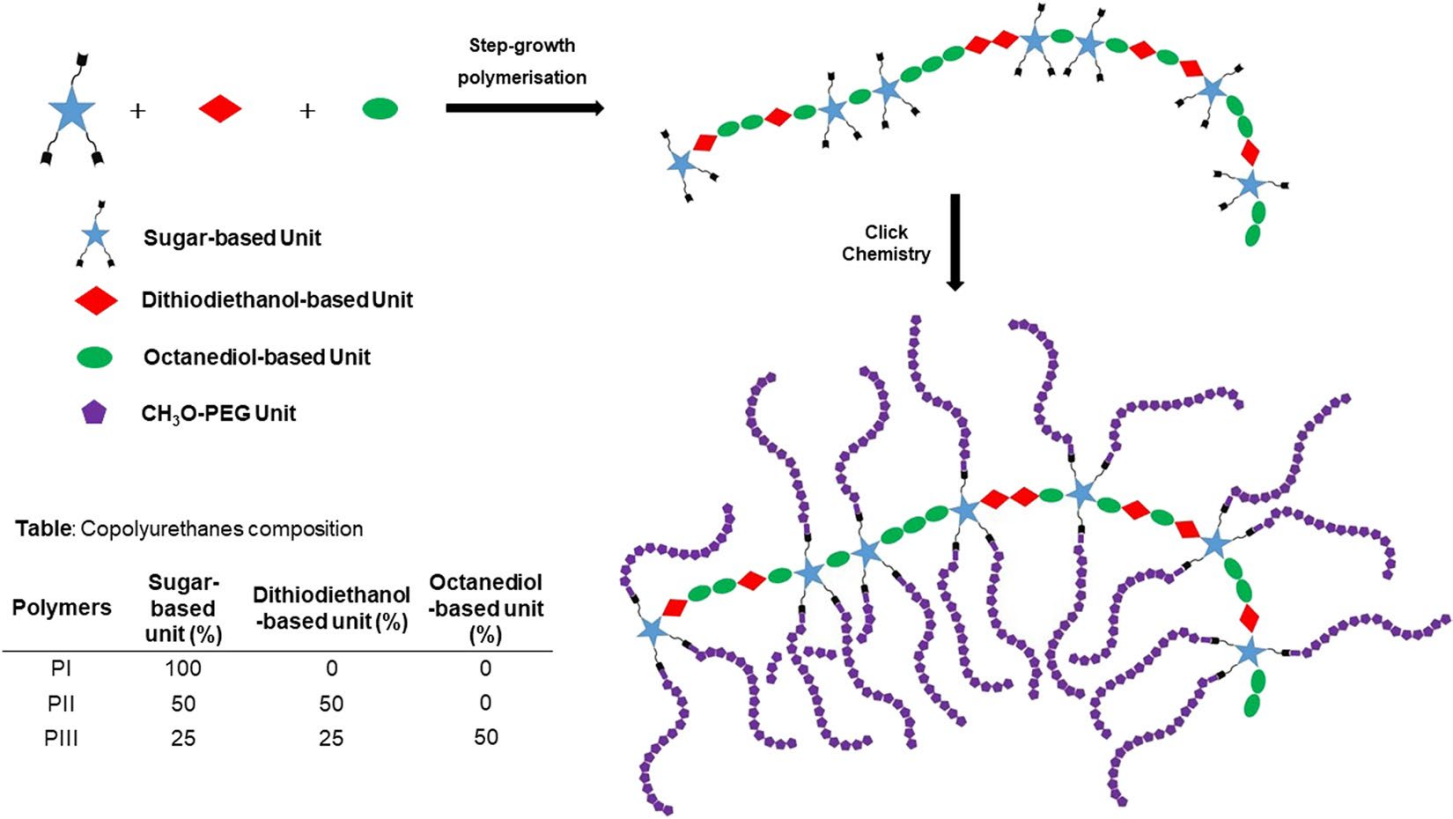
Polymer Synthesis Scheme. PI was constituted only by the sugar-based unit. PII was prepared with a 50% of the sugar-derived unit plus a 50% of the dithiodiethanol-based one. In the PIII copolyurethane, a 25% of the sugar-based unit was used together with a 25% of the dithiodiethanol-derived one plus a 50% of the octanediol-based unit.

All chemicals and solvents were purchased from Aldrich Chemical Co and used without further purification. Polymeric materials were prepared according to the methodology described by our synthesis research group^52^. Polymers were based on three different units which were obtained from hexamethyene diisocyanate (HDI) and a diol to generate the urethane bonding. Diol was selected among tripropargyl-l-arabinitol, dithiodiethanol or 1,8-octanediol. The mixture of the units in different proportions and a subsequent *Click Chemistry* reaction with methoxypolyethylene glycol azide led to the different types of copolyurethanes. Figure 1 shows a schematic of the composition of our three polymers, denoted PI, PII and PIII. In the case of PI, the whole polyurethane was constituted by the sugar-based unit. PII was prepared with a 50% of the sugar-derived unit plus a 50% of the dithiodiethanol-based fraction. However, in the PIII copolyurethane a 25% of the sugar-based unit was used together with a 25% of the dithiodiethanol-derived one plus a 50% of the octanediol-based fraction. Additional information concerning polymers characterization is included in Tables S1 and S2. The compounds were solubilized in dry DMSO, by employing magnetic stirring agitation, to the following concentrations: 5 × 10^−3^ mg/ml for PI and 10^−2^ mg/ml for both PII and PIII. Solubilization time was markedly longer in the case of PI, which took several days to completely redisperse in the final solvent volume. PII and PIII completely dissolved within ten minutes.

The AuNP synthesis was performed by mixing of 10 ml of a 0.25 mM HAuCl_4_ solution in DMSO with 10 ml of each of the three as-prepared polymer solutions under magnetic stirring. Once completely mixed, 1 ml of NaBH_4_ 0.1 M in DMSO was added. Solutions turned brown-red instantly, and were kept stirring at room temperature for 24 h to allow complete reduction of gold salts. Stable gold nanoparticles were formed in all formulations, as evidenced by the appearance of a wide absorbance band around 525 nm. This procedure leads to a 3% gold concentration in SI system and 1.5% gold concentration in SII and SII ones.

### UV-vis absorbance measurements

UV-vis absorbance was characterized in a Cary 500 spectrophotometer at 298 K from 400 to 800 nm. Wavelength accuracy and the spectral bandwidth were ± 0.3 and 0.5 nm, respectively.

### SEM and TEM/HRTEM measurements

SEM experiments were conducted using a SEM-FEG Hitachi S4800 microscope. Prior to the measurements, samples were coated with a Cr layer of approximately 3 nm. For TEM examinations, a single drop (10 μl) of the aqueous solution containing gold nanoparticles was placed on a copper grid coated with a carbon film. The grid was left to dry in air for several hours at room temperature. TEM analysis was carried out in a Hitachi CM 200 electron microscope working at 200 kV. For HRTEM analysis the inkjet printing method was used to deposit the Au-polymers on a carbon coated copper. A FEG high-resolution transmission electron microscope (HRTEM) from FEI Company, USA (model TECNAI G2 F30 S-twin) with the scanning-transmission capabilities (STEM) was used. The experiments were carried out at 300 kV with 0.2 nm point resolution. The microscope was equipped with a high angle annular dark field (HAADF) detector from Fischione Instruments, USA (0.16 nm point resolution), and an INBCA ZX-max 80 silicon drift detector (SDD) for the energy-dispersive X-ray analysis (EDX). The GHR micrograph analysis, lattice spacing, Fast Fourier Transform (FFT) and phase interpretation was performed with the Gatan Digital micrograph software (Gatan Inc., USA) and the Java version of the Electron Microscope Software (JEM). The analysis of the HAAD-STEM images and the EDX spectra profile were conducted with the ES Vision software (FEI Company, USA).

### Material jetting

#### Ink preparation

Inks were prepared from the polymer-encased AuNPs suspensions synthesized as described, using the same amounts of polymer and gold salt. Before injecting in the printer cartridges, suspensions were sonicated for 10 min and filtered through a 5 µm nylon filter to remove potential nanoparticles agglomeration.

#### Printability assessment

Printability tests were carried out in a FujiFilm Dimatix DMP-2800, using cartridges characterized for a 21 µm nozzle diameter with a Drop-on-Demand (DOD) droplet generation. A DOD IJP requires the use of fluid inks with certain characteristics. These characterisctics are revealed in different dimensionless groupings of physical constants, such as the Reynolds, the Weber or the Ohnesorge (Oh) numbers. In 1984, Fromm^61^ described a printability indicator, called the Z parameter, to establish the potencial printability of a given ink. This parameter was defined as Eq. 1:

1 $$Z=\frac{1}{Oh}=\frac{\sqrt{\rho \,l\,\gamma }}{\mu }$$

where *l* is the nozzle diameter and *ρ*, μ and *γ* are the density, dynamic viscosity and surface tension of the fluid, respectively. In 2000, Reis and Derby^62^ used numerical simulation of drop formation to suggest a range of Z within which droplet formation should be stable. This range was set at 1 < *Z* < 10. According to this, Z parameters corresponding to each ink was calculated in order to estimate their potential printability.

A Malvern Kinexus Pro Rheometer equipped with a parallel plate, at 200 µm separation, was used for viscosity measurement under shear rates from 25 s^−1^ and 1000 s^−1^. Each measurement was carried out at 25 °C. At each shear rate, the viscosity was recorded at 5 s intervals within a 180 s test time. For the determination of surface tension a Kruss DSA100S was used, applying the pendant drop method assisted by the Young-Laplace equation^63^. Sample density was measured by weighing 1 ml of each ink.

#### Inkjet printing of the university of seville logo

The University of Seville logo was built with 5 layers of the developed gold nano particle ink formulation with Dimatix printer. The logo image was printed at 35 µm droplet spacing using all 16 nozzles in the Dimatix 10pL printhead. EPSON premium glossy photo paper was used as a substrate, which was heated to 50 °C during the printing process. The printed pattern was further dried with heat gun until the residual liquid was fully evaporated.

### ToF-SIMS experiment

Time-of-flight secondary ion mass spectrometry (ToF-SIMS) analysis of negatively charged secondary ions was carried out with a TOF.SIMS 5 system from ION-TOF GmbH (Münster, Germany) using a 25 keV Bi^3+^ ion beam operated in the high current bunched mode delivering 0.3 pA with 100 μs cycle time, resulting in a mass range between 0 and 875 mass units. Secondary ion maps were acquired using the stage raster mode that stitches several patches of the maximum beam raster area of 500 × 500 μm^2^. The large area scan was performed on an 18 × 18 mm^2^ region containing 1813 × 1813 pixels which comprised of 37 × 37 patches sized 49 × 49 pixels each. For the detailed scan of a letter, the scan was performed on a 2 × 2 mm^2^ region containing 1000 × 1000 pixels. In both analyses, the whole area was scanned once with one shot per pixel and 10 scans per patch, ensuring static conditions. A low energy, 20 eV, electron flood was used to neutralize charge build up on the sample surface. To achieve better contrast, ToF-SIMS mapping data was deionised by performing principal component analysis (PCA) in a dataset containing several ion maps with subsequent data reconstruction retaining only signal-related components. PCA was performed using simsMVA (www.mvatools.com).

## Results and Discussion

### Polymers synthesis

A diverse family of multifunctional polyurethanes has recently been reported by our group^31^ including the synthesis and characterization of new highly biodegradable multipropargyl and *comb*-like copolyurethanes through a combination of step growth polymerization and *Click Chemistry*. These new brush polymers were achieved with complete control of the density, distribution or branch nature; additionally, characteristics related to hydrophilic/lipophilic character were controlled by the selection of the reactants used.

### AuNP-PU interactions

The ultimate goal is to get a stable ink using sugar-based *comb*-like polyurethanes that not only stabilizes the gold nanoparticles, which by themselves are not printable, but also used at a certain concentration to make a colloidal solution of optimal rheological properties to allow it to be used as a printing ink. In this respect, it should be noted that when metallic salt reduction is carried out in a restricted geometry environment, the resulting particles’ size and shape will tend to differ from that of the obtained in a restriction-free medium. This is due, amongst other factors, to the influence of the size of the cavities in which particle growth takes place. In this way, morphological differences between pure solvent-synthesized nanoparticles and those synthesized in a polymer matrix can be indicative of supramolecular structure formation, as well as of the stabilizing properties of the aforementioned polymers. As described in the Experimental Section, gold salt reduction by borohydride was carried out in pure DMSO and with polymers, on a given concentration. These new systems were named in the following way: SI denoting the nanocomposite formed by AuNP and PI, SII denoting the system AuNP-PII and SIII denoting AuNP-PIII.

Absorbance measurements (Fig. S1) of the as-synthesized solutions did show a clear blue-shift of the absorbance band in all cases when compared to the position of the pure borohydride particles. Absorbance spectra for gold-free PI-PIII solutions at the synthesis concentrations can be found in Fig. S2. It is interesting to note that, in the case of SII, whose absorption spectrum is located on the visible region, subtracting the original PII absorbance spectrum did cause negative intensities to appear. This effect is due to a portion of the polymer degrading upon the synthesis procedure. However, the presence of the absorbance bands located at 460 and 482 nm in the uncorrected solution does point to a portion of the polymer remaining in its native form.

**Figure 2:**
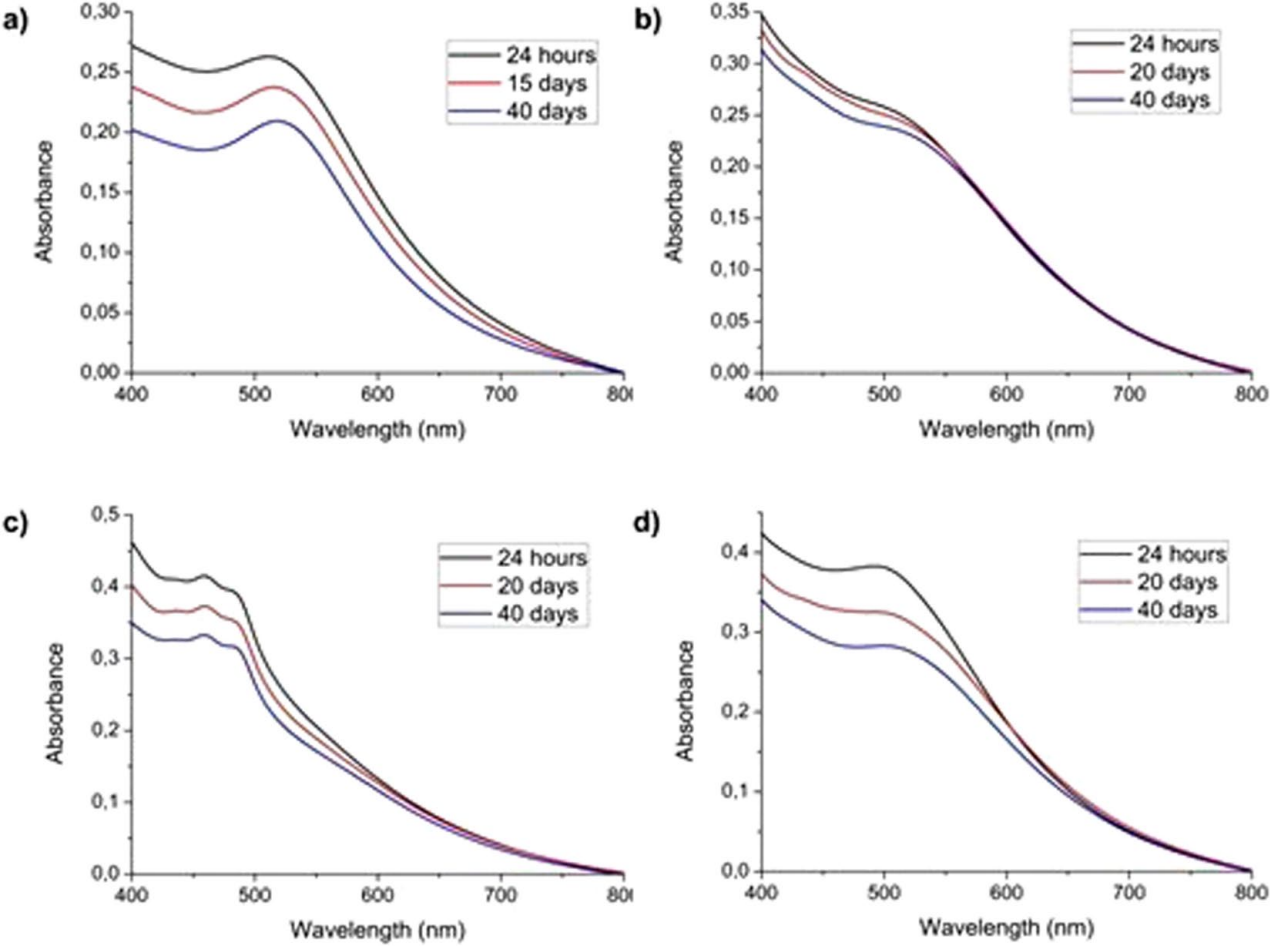
Normalized time-correlated visible absorbance spectra for gold nanoparticle synthesis in the (**a**) absence of polymer (after particle resuspension by stirring), (**b**) SI, (**c**) SII and (**d**) SIII.

Stability measurements were performed for a span of over a month. Results showed no appreciable formation of aggregation bands not only for diluted concentrations, but even for the concentrated original solution during the measured timespan (Fig. 2). An analysis of the stability results can be found in Table S3. In the case of PII, the strong polymer absorbance band did not allow for a clear determination of the absorbance maximum, so the λ_max_ value was employed only in order to allow for the determination of the absorbance loss. A general tendency to red-shifting of the absorbance band, which can be attributed to nanoparticle growth over time, was found in all cases. DMSO borohydride nanoparticles, with no polymers added, were found to deposit in the beaker’s bottom, but they did not aggregate and could be redispersed by stirring prior to measuring. In contrast, polymer-synthesized nanoparticles did not deposit at all, and the solutions remained stable. No gold aggregates were observed on either the polymer-encased or on the free nanoparticles.

In light of the results, the most stable nanoparticles precipitation-wise are those obtained when PI is used as a matrix (8.6% absorbance loss over 40 days), but SI also presents the higher band drift over the studied period (2.2%), which indicates that, even though the gold nanoparticles are resistant to precipitation, they do grow markedly over time, due to residual gold salt reduction. Despite the general particle size increase, no band widening occurs, which is an indicator of the size distribution remaining roughly the same. In contrast, PIII does offer the most protection against particle size changes (1.2% λ_max_ shift) but the obtained colloid is more unstable.

Absorbance measurements highlight that all three polymers are able to stabilize gold nanoparticles in suspension, to a greater or lesser degree. In contrast, polymer-free nanoparticles required resuspension. This means that, while the negatively-charged borohydride is able to interact with the gold surface and prevent aggregation by itself, it is not stable enough in solution to allow the nanoparticles to remain in suspension.

**Figure 3:**
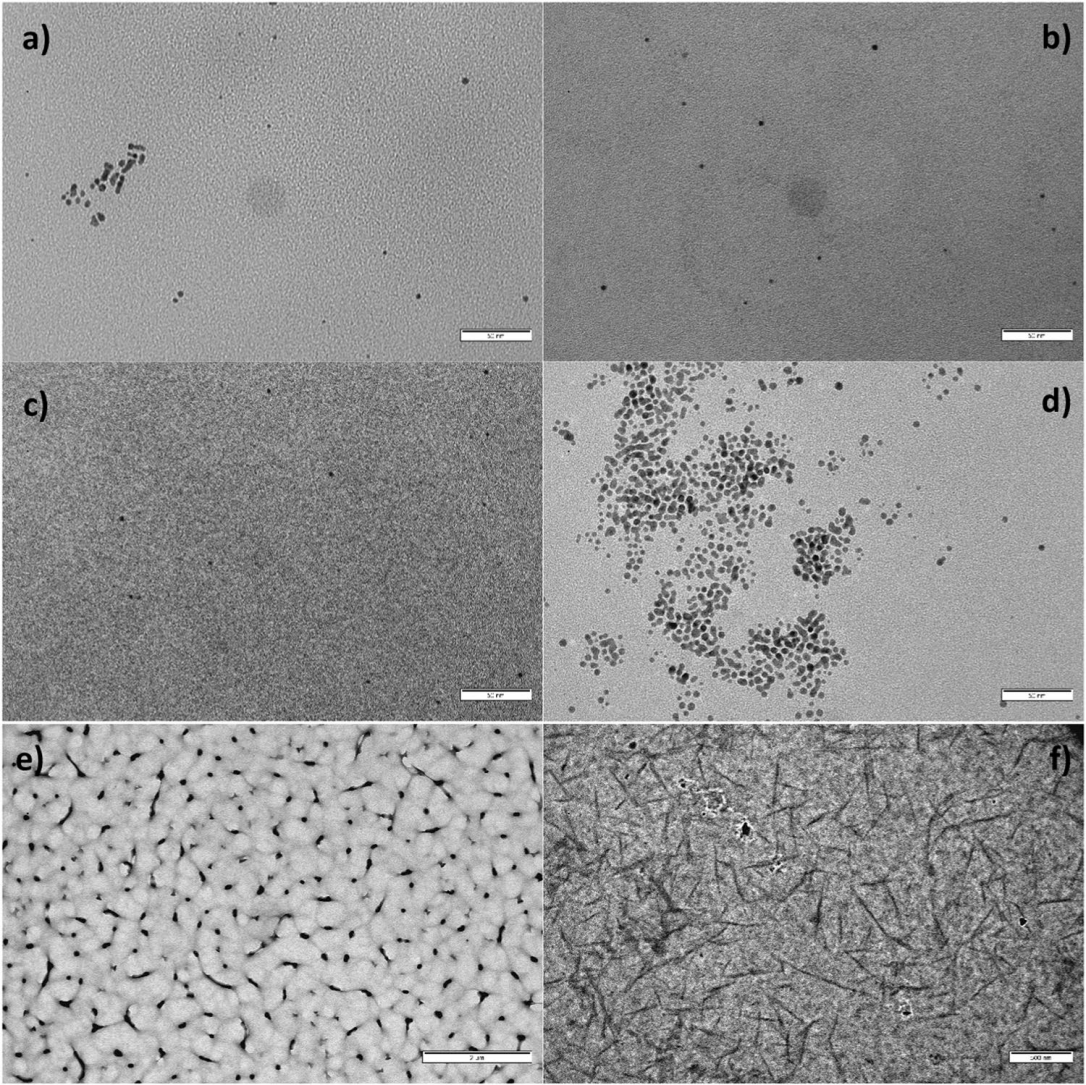
TEM images of (**a**) polymer-free nanoparticles, (**b**) SI, (**c**) SII and (**d**) SIII, the former showing spherical, non-aggregated nanoparticles and the latter showing aggregate morphology and multi-twinned particle formation. Scale bar corresponds to 50 nm in all cases. (**e**) SI polymer structures found upon deposition of the synthesis onto the TEM grids. Scale size corresponds to 2 µm. (**f**) SII polymer structures with a scale size corresponding to 500 nm.

TEM imaging showed that, in all cases, particles obtained in both the presence and absence of the polymers are spherical. No other distinct shapes are formed, although for the unprotected AuNP and SIII some multi-twinned particles appeared (Fig. 3a,d). Those structures are absent in both SI and SII (Fig. 3b,c). The formation of multi-twinned particles is coherent with the polymer-free AuNP and SIII being the two most unstable syntheses. Analysis of the TEM images with ImageJ software yields both the medium particle size and the polydispersity of the samples (Table S4). As all synthesis are over 15% polydispersity, none of them can be considered as monodisperse. However, SIII does present a markedly smaller polydispersity value. It is also interesting to note that for both SI and SII, crystallization of the polymer in different shapes (globular for SI, acicular for SII) can be observed in the TEM grids (Fig. 3e,f). Those structures are completely absent in SIII.

The synthesis was carried out by using borohydride as a reducing agent. This is likely the cause of the small size of the nanoparticles obtained. Size analysis showed that both SI and SII had smaller particle sizes than that of the particles obtained in the absence of polymer. This phenomenon would indicate that the formation of polymer suprastructures has a restricting effect over particle growth.

SI and SII did not present any form of particle coalescence although in both cases a slow growth took place. This indicates that the same superstructures that restrict particle growth are able to keep nanoparticles separated from one another quite effectively, but are not able to stop diffusion of reduced gold atoms to the particle surface. It also evidences that residual reduction of gold salts takes place over a very prolonged period of time. In the case of SIII, restricted growth phenomena were absent and particle size was greater than that of borohydride-protected nanoparticles. The presence of multi-twinned particles in SIII (as shown in Fig. 3b) also indicated a greater degree of mobility of the synthesized nanoparticles, which is coherent with the observed absorbance loss over time. The fact that particle size is actually greater, and not smaller, than that of the polymer-free synthesis pointed to a very different structure of SIII in regards to the other two polymers. Also, for PIII embedded nanoparticles, gold reduction takes place much faster than in the other two’s case, as evidenced by the fact that almost no residual particle growth takes place over time despite the lack of space restrictions. For future applications, some avenues to improve both stability and shelf-life of the synthesized colloids could be explored: for example, dialysis of the colloid suspensions on 1 L of fresh DMSO, using frequent solvent changes, over a period of 48–72 h could prove useful to reduce the concentration of both residual gold salts and borohydride on AuNPs synthesis.

### Material jetting of AuNP inks

**Figure 4:**
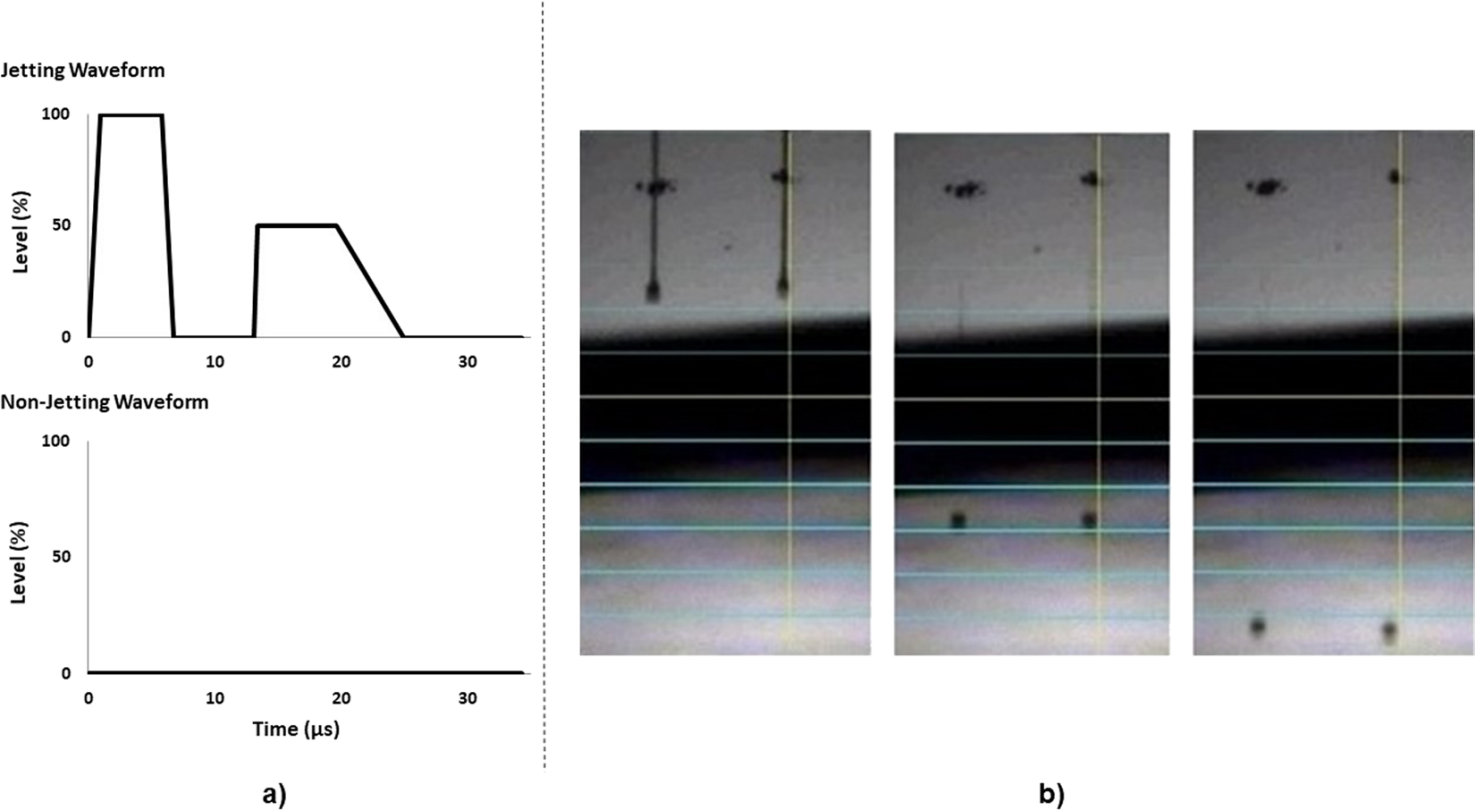
(**a**) Complex jetting waveform used for printing the three inks. (**b**) Sequence of droplet formation of ink SIII using the complex waveform displayed in a).

As a first approach to check the potential inkjet printability of the polymer-encased AuNP, inks were prepared exactly as the suspensions of polymer-AuNP described in the Experimental Section. Viscosity data for the different inks at 25 °C are shown in Fig. S3 and Table 1. Inks SI, SII and SIII presented viscosity values of 1.87 × 10^−3^ ± 3 × 10^−5^ Pa·s, 2.02 × 10^−3^ ± 3 × 10^−5^ Pa·s and 2.09 × 10^−3^ ± 2 × 10^−5^ Pa·s, respectively. These values were in concordance with the variation in the polymers concentrations and their increment in Mw in each ink. However, manufacturer’s guideline for the use of Dimatix DMP-2800 suggests an ideal ink viscosity around 1 × 10^−2^ Pa·s, which is higher than the values obtained for the inks. Thus, the printing temperature was set at 25 °C. Surface tension values (Table 1) were 44.62 ± 0.38, 41.19 ± 0.14 and 41.79 ± 0.09 mN/m for inks SI, SII and SIII, respectively. The addition of such a small amount of polymer and gold salts to each ink has a negligible effect on the density values when compared to the solvent density. Using Eq. 1, the Z parameter obtained for inks SI, SII and SIII were 16.9, 15.4 and 14.8, respectively. These values were outside the ideal range for a stable droplet formation established by Reis and Derby^62^. However, a reliable droplet generation was achieved through the adoption of a complex jetting waveform (Fig. 4a). As an example of the stability of the drop formation achieved using this complex waveform during the printing, Fig. 4b displays a sequence of the droplet generation of ink SIII.

**Table 1:** Values of shear viscosity, surface tension and density and Z parameter for each ink.

Ink	Shear Viscosity (mPa·s)	Surface Tension (mN/m)	Density (mg/ml)	Z Parameter
SI	1.87 ± 0.03	44.62 ± 0.38	1.1	16.9
SII	2.02 ± 0.03	41.19 ± 0.14	1.1	15.4
SIII	2.09 ± 0.03	41.79 ± 0.09	1.1	14.8

**Figure 5:**
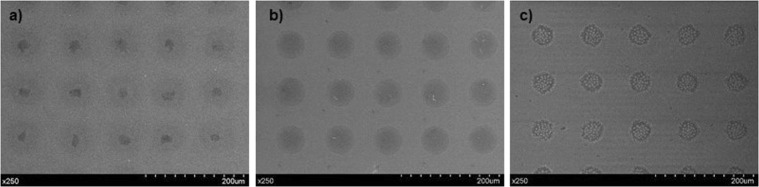
Drops of inks (**a**) SI, (**b**) SII and (**c**) SIII printed on glass slides.

After optimising the inks’ printability, ensuring a stable droplet generation, they were printed on top of glass slides setting 100 µm of drop spacing. Samples, previously coated with a Cr layer, were observed by SEM (Fig. 5). A processing with ImageJ produced average diameters of 33.8 ± 0.8, 29.4 ± 0.2 and 24.6 ± 1 µm for drops obtained from inks SI, SII and SIII, respectively. SEM images also showed a more homogenous spreading distribution of SII droplets when compared to the SI and SIII drying patterns.

**Figure 6:**
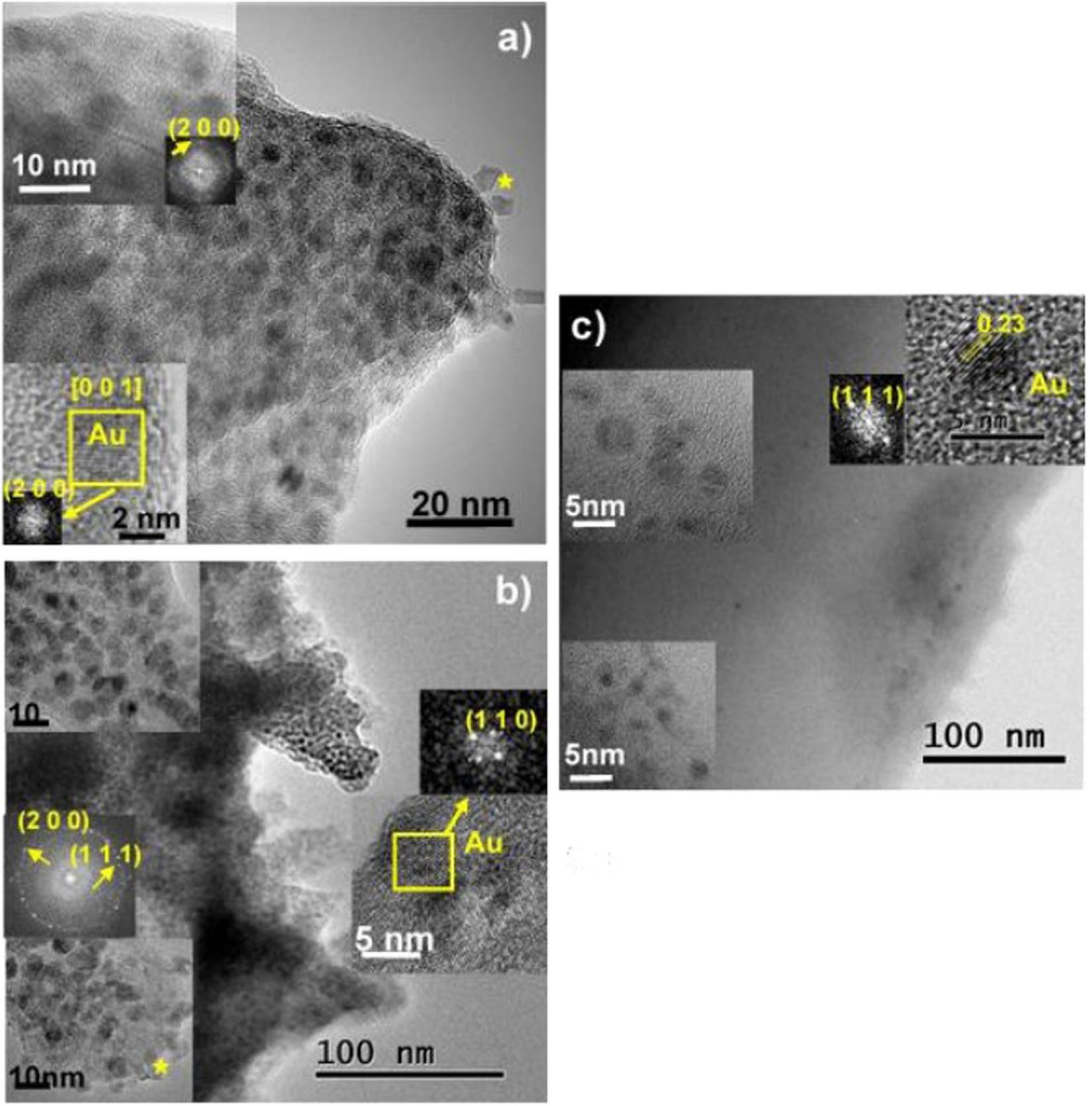
Microcharacterization results of (**a**) SI, (**b**) SII and (**c**) SIII inks, TEM images and HRTEM micrographs (inset) showing the gold nanoparticles and the corresponding FFT with the Au (h k l) planes yellow marked.

**Figure 7:**
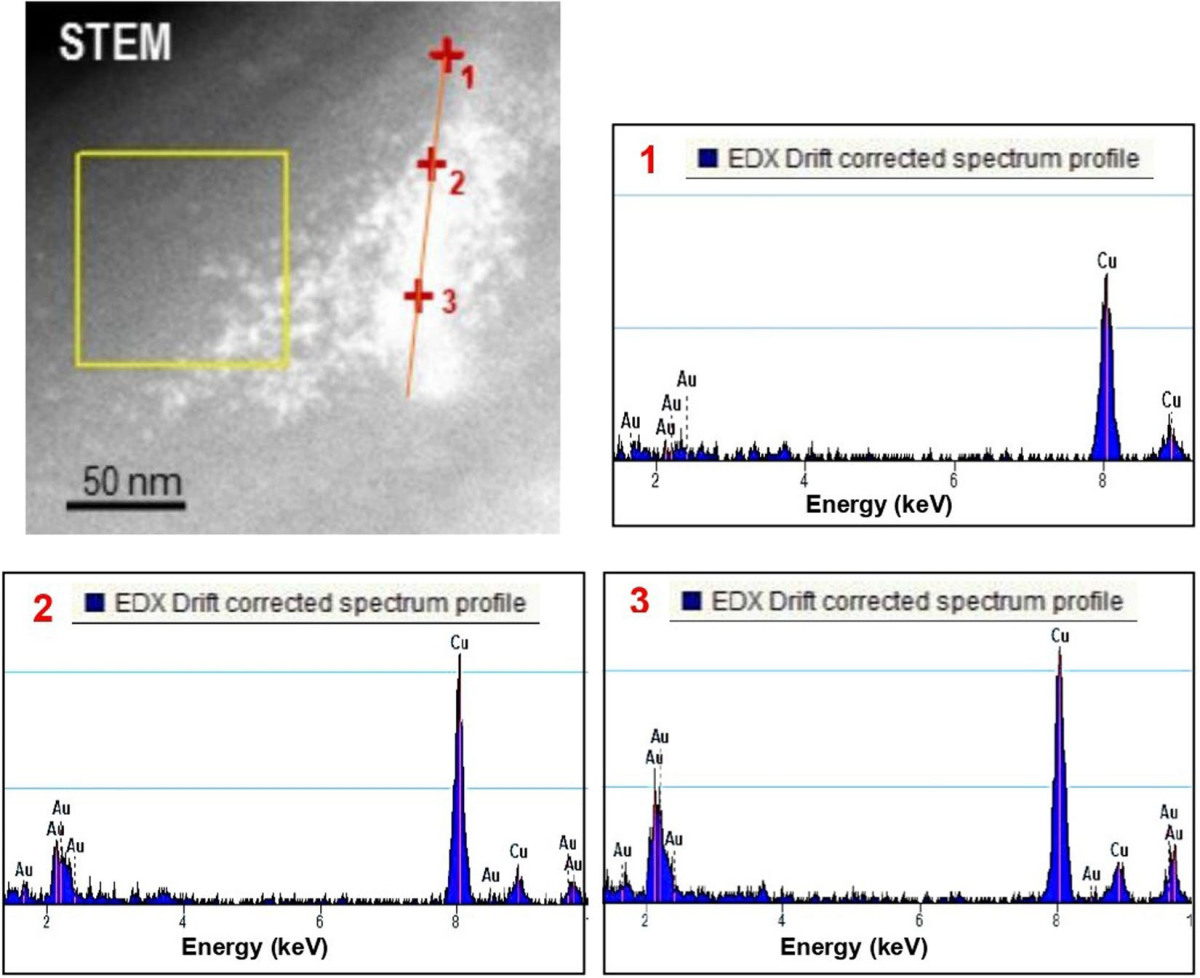
STEM image profile analysis and three EDS spectra taken with a HAADF detector in three different points showing the increase of gold content along the profile.

In order to go further with the micro characterization of the jetted of AuNP inks HRTEM/HAADF/EDS techniques were used and the obtained results corresponding to SI, SII and SIII are depicted in Figs 6 and 7. Systems SI and SII are quite similar and enclose a large agglomeration of gold nanoparticles as can be seen in Fig. 6a,b, respectively. In the high-resolution micrographs that are inset, the crystalline structure of the gold nanoparticles can be observed; its average size is between 5 and 10 nm. These results are in concordance with the previous absorbance measurements, in which a growth of the AuNP diameter was observed with time, thus after six months of storage, gold particles have increased their size from approximately 3 nm up to 5-10 nm. Most of the gold nanoparticles have a rounded shape, nevertheless at the edges of the polymer in some areas they are crystalized with polygonal shape (marked with an asterisk in both polymers). We performed Fast Fourier Transform of selected areas of the collected HRTEM images to determine whether the structures were crystalline. These indicate that the gold has crystallized in the cubic system and spatial group Fm-3m with a = 0.41 nm, the index (h k l) of planes found in the FFT are marked in yellow. Agglomerate SIII contains less agglomeration of gold nanoparticles as can be seen in the TEM image presented in Fig. 7c. In this case, the average size of the gold nanoparticles between 2 and 5 nm, that have been measured in the HRTEM images (inset). These measurements demonstrate that the AuNP size for SIII is constant with time and no particle growth is observed. In the upper right corner, a gold nanoparticle less than 5 nm is clearly visible, where the spacing of 0.23 nm corresponding to the gold planes (1 1 1) can be observed.

In the three systems, chemical analysis was performed by X-ray emission spectroscopy (EDS) in STEM mode. The gold peak was found in all areas where nanoparticles were found in the HRTEM images. In a representative way, the results of agglomerate SIII are presented in Fig. 7, showing a STEM image where the composition has been analyzed along a line profile (marked in red). The EDS spectra collected in points 1, 2 and 3 of the profile indicate how the gold content increases throughout the profile.

**Figure 8:**
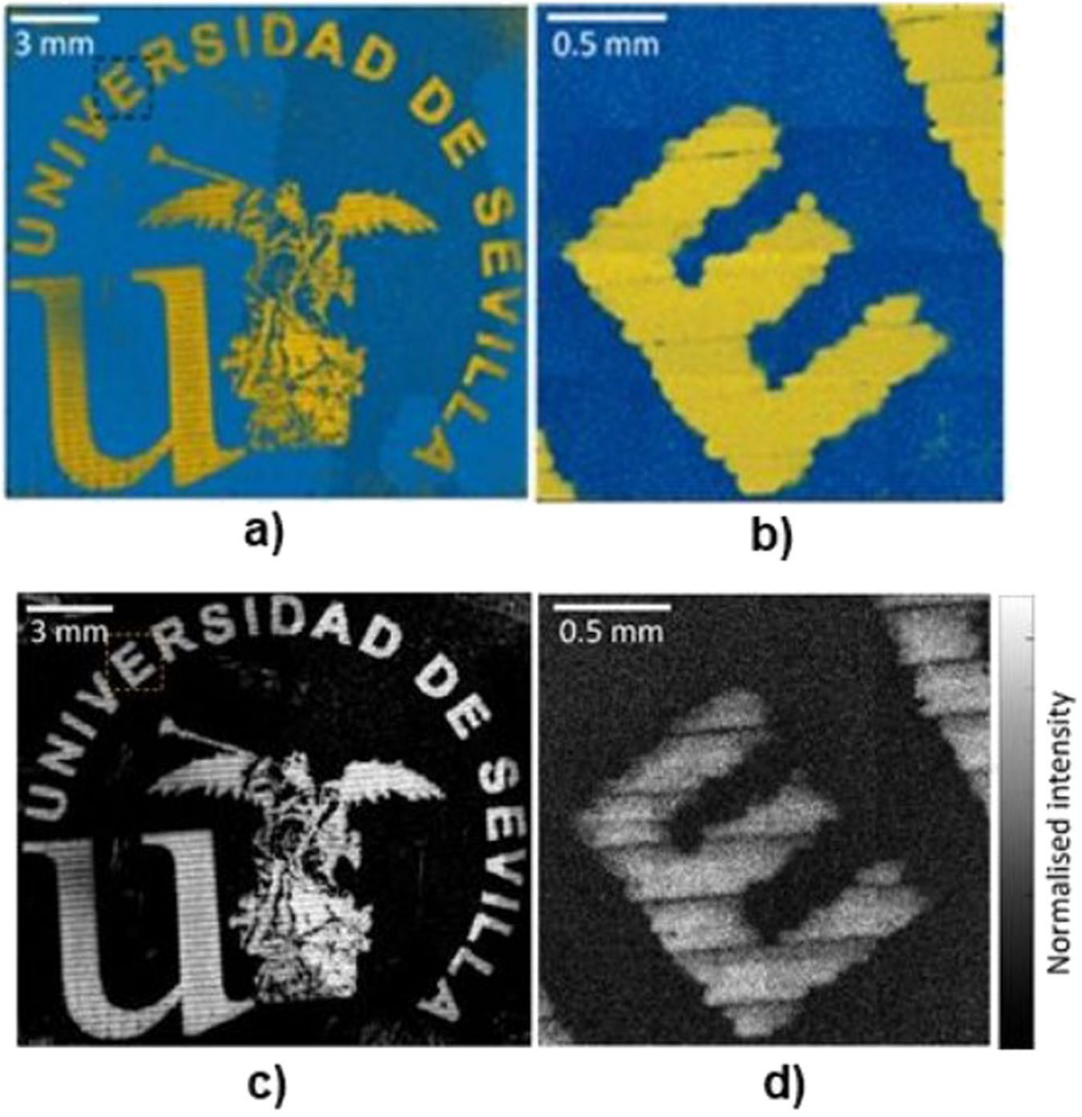
Overlay of ToF-SIMS map of combined ions related to gold (yellow) and the polymer substrate (blue). The signal intensity is proportional to the opacity of each colour. Ions related to gold: AuO^−^, AuOH^−^, AuOH^2−^, AuO^2−^, AuO_2_H^2−^, Au^2−^, Au_2_H^−^, Au_2_H^3−^, Au_2_O^−^, Au_2_H^4−^, Au_2_OH^−^, Au^3−^, Au_3_H^−^ and Au^4−^. Ions related to substrate: C^2−^, C_2_H^−^. (**a**) Large area scan of whole print region. (**b**) Close up scan on a letter “E”. (**c**,**d**) are an alternative version containing only Au signal (no substrate overlay).

According to the results obtained from the stability analysis and the printability test, SII showed a better balance of properties focused toward its usage in MJ Printing, so it has been selected as the most promising ink for further investigation. Subsequently, the University of Seville logo was inkjet printed using SII as the ink, following the methodology detailed in the Experimental Section. Figure 8 shows an overlay of the ToF-SIMS maps of combined ions related to gold (in yellow) and the polymer substrate (blue). The Au^−^ and AuH^−^ were excluded due to interference with the background signal. Various gold-sulphur complexes were also observed in the same area as the regular Au signal (AuS^−^, AuSH^−^, Au_2_S^−^ Au_3_S^−^, etc…). These are representative of the interaction between the gold nanoparticles and the polymer disulfide bridges and thus also indicate the presence of gold.

## Conclusions

Biodegradable *comb*-like copolyurethanes derived from carbohydrates have been investigated as stabilizers for the preparation of long-lasting suspensions of AuNP. Their complete control of the density, distribution and branch nature and its hydrophilic/lipophilic character, together with their biodegradability, made them ideal candidates for their usage as AuNP protectors to form nanocomposites. With the objective of obtaining noble metallic materials that can be employed as inks in inkjet printing, the study of the interactions between AuNP and the three polymers was carried out. Results showed that polymer-synthesized nanoparticles (PU-AuNP) remained stable in suspension, with no aggregates being found. All three polymers were able to stabilize gold nanoparticles in suspension, however polymer-free nanoparticles required resuspension. TEM imaging on fresh samples showed that, in all cases, particles obtained are spherical and size analysis displayed that both SI and SII had smaller particle sizes than that of the particles obtained for SIII. The three systems were tested as inks for MJ Printing after a storage period of six months. All the inks were printable at room temperature through the use of a complex waveform, showing a high stability in the printing process. TEM studies of the printed samples confirmed all AuNP had spherical shape, however, particle size increased from approximately 3 nm up to 5-10 nm for inks SI and SII. No diameter growth was shown for SIII. SEM studies displayed a more homogenous spreading distribution in the case of SII. Compiling all previous results, SII was selected as the most promising ink for its utilization in a IJP process. Thus, this system was used to print the University of Seville logo. ToF-SIMS experiments demonstrated the presence of Au in the printed pattern, as well as the linkage Au-S, confirming the interaction between the particle and the sulfur atom from the polymer disulfide bond as part of the stabilization process of the AuNP. Considering the success in the utilization of these *comb*-like copolyurethanes as AuNP stabilizers and their use as inks for MJ printing, the increment of the Au/polymer ratio in these systems, especially in the case of SII, will be conducted in our laboratory in due course.

## Supplementary information

Supplementary information
