# Author Correction: Design of highly stabilized nanocomposite inks based on biodegradable polymer-matrix and gold nanoparticles for Inkjet Printing

**DOI:** 10.1038/s41598-020-76435-1

**Published:** 2020-11-13

**Authors:** Belen Begines, Ana Alcudia, Raul Aguilera-Velazquez, Guillermo Martinez, Yinfeng He, Gustavo F. Trindade, Ricky Wildman, Maria-Jesus Sayagues, Aila Jimenez-Ruiz, Rafael Prado-Gotor

**Affiliations:** 1 Department of Organic and Medicinal Chemistry, School of Pharmacy, University of Seville, Seville, 41012 Spain; 2 Centre for Additive Manufacturing, Faculty of Engineering, University of Nottingham, Nottingham, NG7 2RD UK; 3 Advanced Materials and Healthcare Technologies, School of Pharmacy, The University of Nottingham, University Park, Nottingham NG7 2RD UK; 4 Material Science Institute of Seville, CSIC/US, Seville, 41092 Spain; 5 Department of Physical Chemistry, School of Pharmacy, University of Seville, Seville, 41012 Spain

Correction to: *Scientific Reports* 10.1038/s41598-019-52314-2, published online 06 November 2019

Gustavo F. Trindade was omitted from the author list in the original version of this Article. This has been corrected in the PDF and HTML versions of the Article, and in the accompanying Supplementary Information file.

The Author Contributions section now reads:

A.A. and R.P.-G. envisaged the work to use biodegradable and biocompatible polymers recently described by our group to be used as stabilizer of gold nanoparticles for potential biomedical devices. B.B., R.A.-V. and G.M. scaled up the synthesis to obtain enough amounts of polymers with minor changes considering the previous published synthesis, while A.J.-R. prepared and tested the stability of the AuNP systems to collect data to difference among the metallic nanoparticles, Y.H. designed, fulfilled and characterized all the parameters necessary for the inkjet printing strategy. G.F.T. characterized and analyzed the printed specimen through ToF-SIMS. M.-J.S. carried out the TEM microscopy characterization. B.B. and A.J.-R. were primarily in charge of compiling all the experimental data to write the manuscript. Besides, A.A., Y.H., R.W., M.-J.S. and R.P.-G. proofread it.

The Acknowledgements section now reads:

This work was supported by the Engineering and Physical Sciences Research Council (grant number EP/N024818/1, EP/P031684/1), by the Spanish Ministerio de Economía y Competitividad MinECo, (Grants Nos. CTQ2016-78703-P and MAT2016-78703-P), by the Junta de Andalucía (Consolidation Grant for Research Group FQM-135 and 2017/FQM-386, P-2018/809) and the University of Seville (V y VI Plan Propio PP2016-5937 and OTRI 2010/00000762). The European Union through the CT-2011-1-REGPOT285895 AL-NANOFUN project for the microscopy facilities sited in Seville is also gratefully acknowledged. Authors would like to thank the Functional Characterization and NMR Services from CITIUS, University of Seville, for the support offered to develop this work and Dr. Manuel Pernía for reviewing the draft.

