# Supplementary material for: Design of highly stabilized nanocomposite inks based on biodegradable polymer-matrix and gold nanoparticles for Inkjet Printing
Source: Sci Rep. 2019 Nov 6;9:16097. doi: 10.1038/s41598-019-52314-2 (PMC6834569; doi:10.1038/s41598-019-52314-2)
Supplement: Supplementary file 1 — Supplementary information [file 41598_2019_52314_MOESM1_ESM.docx]

**Design of highly stabilized nanocomposite inks based on biodegradable polymer-matrix and gold nanoparticles for Inkjet Printing**

**Belen Begines^1,+^, Ana Alcudia^1^, Raul Aguilera-Velazquez^1^, Guillermo Martinez^1^, Yinfeng He^2^, Ricky Wildman^2^, Maria‑Jesus Sayagues^3^, Aila Jimenez-Ruiz^4,+,*^, Rafael Prado-Gotor^4,*^**

^1^Department of Organic and Medicinal Chemistry, School of Pharmacy, University of Seville. Seville, 41012. Spain

^2^Centre for Additive Manufacturing, Faculty of Engineering. University of Nottingham. Nottingham, NG7 2RD. United Kingdom

^3^Material Science Institute of Seville, CSIC/US. Seville, 41092. Spain

^4^Department of Physical Chemistry, School of Pharmacy, University of Seville. Seville, 41012. Spain

*** To whom correspondence should be addressed. Email: [ajimenez28@us.es](mailto:ajimenez28@us.es), [pradogotor@us.es](mailto:pradogotor@us.es)

+ These authors contributed equally to this work


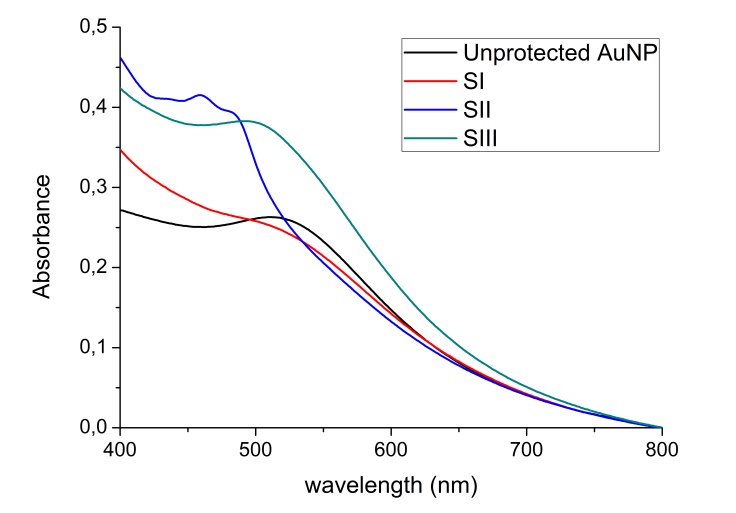


**Figure S1**. Absorbance measurements of the gold nanoparticles obtained by gold salt reduction in the presence and absence of polymers.


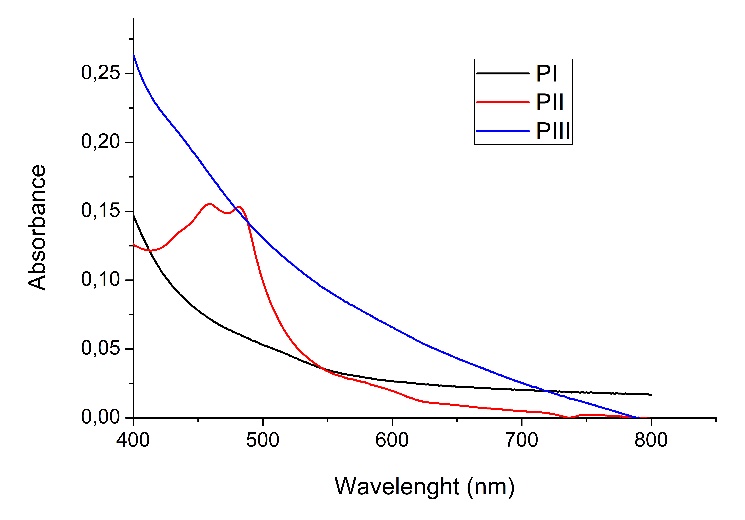


**Figure S2**. Absorbance spectra of gold-free PI-PIII solutions at the synthesis concentrations


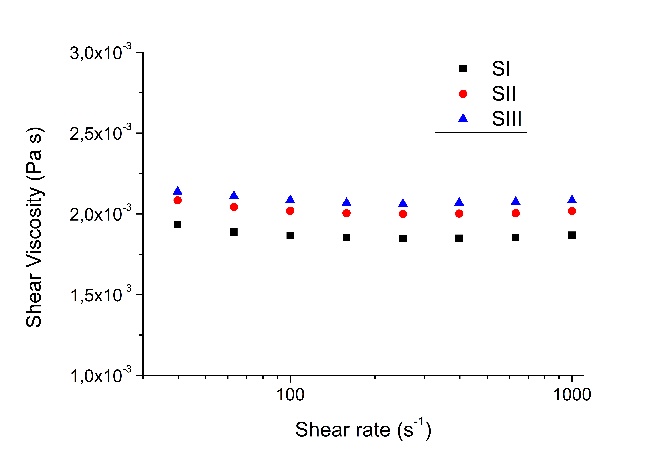


**Figure S3.** Viscosity data as a function of shear rate for each ink.

| **Polymer** | **M_n_ (Da)** | **M_w_ (Da)** | **M_n_/M_w_** |
| --- | --- | --- | --- |
| PI | 24300 | 55100 | 2.3 |
| PII | 27700 | 60500 | 2.2 |
| PIII | 23100 | 48200 | 2.1 |

**Table S1:** Molecular weights and polydispersities of polymers, both determined by GPC.

| **Polymers** | **T*_g_* (°C)^a^** | **T*_m_* (°C)^a^** | **Δ*H_m_* (J/g)^a^** | **°*T_d_* (°C)^b^** | **^max^*T_d_* (°C)^b^** | **ΔW (%)^b^** |
| --- | --- | --- | --- | --- | --- | --- |
| PI | 10 | 21 | 33 | 309 | 264/394 | 8/82 |
| PII | -1 | 18/118 | 8/11 | 300 | 271/335/393 | 7/20/64 |
| PIII | 6/68 | 21/129 | 31/14 | 303 | 297/347/394/449 | 9/28/58/2 |

**Table S2:** Thermal properties of polymers. ^a^ Glass transition temperature (T*_g_*), melting temperature (T*_m_*) and enthalpy (Δ*H_m_*) measured by DSC; ^b^ Onset decomposition temperature corresponding to 10% of weight loss (°T*_d_*), maximum rate decomposition temperatures (^max^T*_d_*) and weight loss at the respective decomposition step [ΔW(%)] determined by TGA.

| **System** | **Initial λ_max_ (nm)** | **Initial absorbance** | **Final λ_max_ (nm)** | **Final absorbance** | **% λ_max_ drift (40 days)** | **% Abs loss (40 days)** |
| --- | --- | --- | --- | --- | --- | --- |
| AuNP  (no polymer) | 511 | 0.263 | 519 | 0.209 | 1.6% | 20.5% |
| SI | 505 | 0.256 | 516 | 0.234 | 2.1% | 9.4% |
| SII | *-* | 0.415 | *-* | 0.333 | - | 19.8% |
| SIII | 493 | 0.383 | 499 | 0.283 | 1.2% | 35.3% |

**Table S3.** Normalized absorbance band intensities and positions over the measured time spans

| **System** | **Particle size (nm)** | **Polydispersity** |
| --- | --- | --- |
| AuNP (no polymer) | 3.2 ± 1.0 | 33% |
| SI | 2.7 ± 0.7 | 25% |
| SII | 2.3 ± 0.7 | 30% |
| SIII | 4.2 ± 0.6 | 16% |

**Table S4.** Particle size and polydispersity values obtained through TEM image analysis
